# Auditory Cortex Asymmetry Associations with Individual Differences in Language and Cognition

**DOI:** 10.3390/brainsci14010014

**Published:** 2023-12-23

**Authors:** Mark A. Eckert, Kenneth I. Vaden, Silvia Paracchini

**Affiliations:** 1 Department of Otolaryngology—Head and Neck Surgery, Medical University of South Carolina, Charleston, SC 29425, USA; vaden@musc.edu; 2 School of Medicine, University of St. Andrews, North Haugh, St. Andrews KY16 9TF, UK; sp58@st-andrews.ac.uk

**Keywords:** cerebral lateralization, planum temporal asymmetry, reading disability, language impairment

## Abstract

A longstanding cerebral lateralization hypothesis predicts that disrupted development of typical leftward structural asymmetry of auditory cortex explains why children have problems learning to read. Small sample sizes and small effects, potential sex-specific effects, and associations that are limited to specific dimensions of language are thought to have contributed inconsistent results. The large ABCD study dataset (baseline visit: N = 11,859) was used to test the hypothesis of significant associations between surface area asymmetry of auditory cortex and receptive vocabulary performance across boys and girls, as well as an oral word reading effect that was specific to boys. The results provide modest support (Cohen’s *d* effect sizes ≤ 0.10) for the cerebral lateralization hypothesis.

## 1. Introduction

Landmark case studies linking a history of written and oral language learning problems to atypical planum temporale asymmetry (PTA) [1] provided an empirical foundation for a cerebral lateralization hypothesis [2] that reading disability develops from atypical asymmetry of this cortical region that represents acoustic features of speech [3]. Attempts to replicate this observation have been inconsistent, owing to small sample sizes, varied measurement and sampling methods, potential sex effects, and the possibility that the effect size for a PTA and reading disability association is small [4,5]. There remains uncertainty about the extent to which PTA can explain written and oral language problems.

Limited replication may also have occurred if atypical PTA is predictive of language abilities in general and not specific dimensions of language, such as phonological processing. For example, more rightward PTA has been observed in people whose reading skills were expectedly low because of low performance for their language or verbal ability [6], people with developmental language disorder [7], and lower verbal ability in an individual difference study of healthy adolescents and adults [8].

Verbal abilities do not always explain PTA findings in reading disability studies. For example, more rightward PTA was observed in boys with reading disability [9], and while verbal ability differed between reading groups in that study, verbal ability was not significantly associated with PTA. That is, it remains unclear whether PTA relates to specific dimensions of language, language more generally, or a variety of cognitive functions that contribute to estimates of general intelligence.

The current study was designed to address the sample size limitations of previous PTA studies by examining PTA associations with language-related measures in the large sample from the ABCD study [10] (data release version 4; available via the NIMH Data Archive). While the ABCD study has not included deep phenotyping of oral and written language abilities, real word reading and verbal ability measures are available from this sample of more than 11,000 participants. As such, the ABCD study can provide high confidence in the generalizability of findings, even where small effects are concerned. We tested the hypothesis that PTA is specifically associated with language-related measures compared to other cognitive constructs, including executive function, which can be affected in people with language impairment [11] and reading disability [12].

We also examined the degree to which PTA support for the cerebral lateralization hypothesis depended on boys compared to girls in the ABCD sample, as suggested by those landmark case studies [1] and two of the PTA studies described earlier [6,9]. Prenatal testosterone exposure has been hypothesized to impact PTA by limiting neuronal loss in people with symmetrical plana (e.g., limit pruning) [13], with perhaps more pronounced and widespread effects on brain structure when there is perinatal injury, as suggested by the study of rodents with induced perinatal lesions [14]. Meta-analysis has demonstrated limited support for the androgen component of the cerebral lateralization hypothesis [15], however, and brain structure differences between males and females often depend on brain size effects [16]. Nonetheless, a stronger PTA association with language measures in males compared to females is possible, including because of different trajectories of oral and written language development that are reflected in the increased variation for language outcomes among males compared to females [17] and sex and/or gender differences in brain morphology [18,19], including surface area asymmetries [20] that were examined in the current study.

## 2. Materials and Methods

### 2.1. Participants

This study was designed to examine the baseline ABCD sample (data release version 4) from 11,859 children (107 to 133 months of age; girls: 5675; boys: 6184). Results were replicated using data from the 10,416 of these children who were studied approximately two years after the baseline visit (127 to 166 months of age; girls: 4904; boys: 5404). This epidemiological sample was recruited from public schools by personnel at 21 different data collection sites with the goal of accurately reflecting the diversity of race, ethnicity, sex, socioeconomic status, and urbanicity across the United States of America [21]. Exclusion criteria included non-fluent English speaker, magnetic resonance imaging (MRI) contraindication, neurologic disorder, premature birth (<28 weeks) or a birth weight less than 1200 g, birth complications that required hospitalization for more than one month, uncorrected vision, diagnoses of schizophrenia, autism spectrum disorder, intellectual disability, or alcohol/substance use disorder.

The biomedical ethics and clinical oversight guidelines for the ABCD study were established by an ABCD Bioethics and Medical Oversight Advisory Group [22]. Most of the ABCD study sites relied on a central Institutional Review Board (IRB) at the University of California, San Diego, for review and approval of the study protocol. A subset of research sites obtained approval from their local IRB [23]. Informed consent was obtained from a parent/caregiver, and all participants provided assent.

### 2.2. Behavioral Data

The NIH Toolbox Cognition Battery [24] is a widely used assessment tool and was used in the current study to examine the extent to which cognitive variables were related to PTA. This study included a focus on receptive vocabulary and oral real word reading. The receptive vocabulary task requires ~5 min to assess the ability to identify one of four pictures that most accurately represents an aurally presented object, action, or concept word. The oral word reading task requires ~4 min to assess the ability to pronounce single words that are displayed on a computer screen. Participants were allowed to take as much time as necessary to make a response. These word reading items were selected during the development of the NIH Toolbox to have a broad range of reading difficulty and included words with irregular orthography. The receptive vocabulary and word reading tasks were administered until a 0.3 standard error level of accuracy was obtained, or all 25 items from each test were presented. Both tests have high test–retest reliability, as well as high convergent and discriminant validity [25]. The attention (Flanker inhibitory control), executive function (set-shifting), episodic memory (picture sequence memory), processing speed (pattern comparison), and working memory (word list storage capacity) tasks from the NIH Toolbox were also examined to determine the cognitive construct specificity of reading and receptive vocabulary results.

### 2.3. Imaging Data

T1-weighted images were collected across ABCD study sites using General Electric (Chicago, IL, USA), Phillips (Amsterdam, the Netherlands), and Siemens (Erlanger, Germany) 3T systems. The sequence information for each imaging platform is described in [26]. The T1-weighted images were processed with Freesurfer (v5.3.0, Charlestown, MA, USA) [27,28] to obtain regional surface area measures, as defined with the Destrieux atlas [29]. In addition to empirical and theoretical motivations for focusing on planum temporale surface area, these Freesurfer surface area estimates have good multi-site test–retest reliability [30]. All 74 Destrieux regions of interest collected in the ABCD study were examined, with an a priori focus on regions of interest covering the space of the planum temporale. This includes the medial, anterior, and posterolateral regions of the planum temporale (Destrieux labels: Lat_Fis-post, S_temporal_transverse_L, G_temp_sup-Plan_tempo_L, respectively; as shown in Supplementary Figure S1 and later in Results), which allowed for the assessment of whether reading and/or receptive vocabulary associations were stronger for specific planum regions. Surface area asymmetry was calculated with the following standard formula that controls for differences in the overall surface area: (left − right)/((left + right)/2).

### 2.4. Statistics

Descriptive statistics are presented to describe the demographic, behavioral, and brain structure features of the dataset. Because of a focus on the degree to which PTA and behavioral associations were dependent on sex, tests of variance and mean differences between boys and girls were performed. In addition, one-sample t-tests were used to characterize the extent to which participants exhibited significant PTA.

A series of multiple regressions were also performed to test the overarching hypothesis that more leftward PTA predicts better language-related performance. These analyses included statistical controls for sex (and PT interaction), age, parental education, and handedness [31]. There also were variables included in the regression models to control for image quality and multisite differences in scanner hardware and software (Freesurfer topological defects, MRI scanner serial number), which can contribute to differences in structural imaging metrics across sites [30]. We considered the specificity of effects for PTA relative to total cerebral surface area and the specificity of effects for oral word reading relative to receptive vocabulary. We also used multiple regression to examine the specificity of receptive vocabulary associations with PTA relative to the other NIH Toolbox Cognition measures.

Bonferroni correction was used when evaluating the significance of the statistical comparisons in this study. For example, a Bonferroni corrected *p* value (alpha = 0.00238) was used as a threshold for statistical significance across the 21 associations between the 7 toolbox measures and the 3 PTA measures.

## 3. Results

Descriptive statistics are presented in Table 1 for the Time 1 cognitive performance and brain structure measures for boys and girls. This table demonstrates significantly more rightward PTA and lower total surface area in girls compared to boys, for example. The table also highlights the measures for which the boys exhibited significantly more variability than the girls, including the oral word reading, medial PTA, and total surface area measures. Supplementary Table S1 presents the associations between the demographic, receptive vocabulary, oral word reading, and brain structure measures.

### 3.1. Surface Area Asymmetries and Language

**Figure 1:**
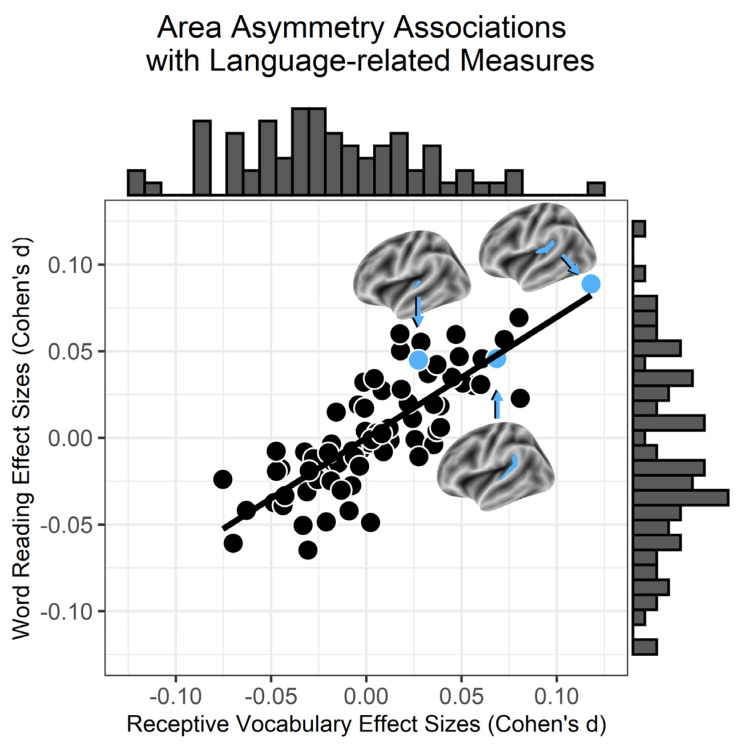
Consistent receptive vocabulary and word reading associations with surface area asymmetries (*r*_72_ = 0.80, *p* < 2.2^−16^). Each symbol is a different Destrieux region of interest (ROI). Cohen’s *d* effect sizes are shown for the associations between each ROI and the receptive vocabulary and word reading measures after adjusting for covariates. Arrows point from a PTA region of interest to the corresponding symbol in blue. A listing of effect sizes for each region of interest is reported in Supplementary Table S2.

**Figure 2:**
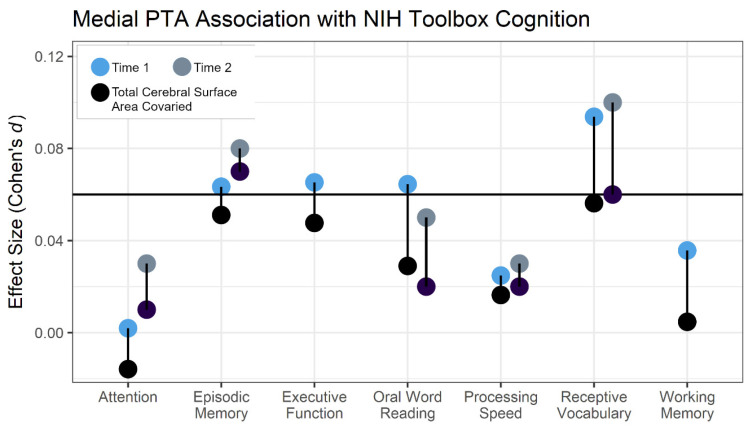
Effect sizes for the relationship between medial PTA and NIH Toolbox Cognition measures are shown before (blue circles: baseline Time 1 visit; gray circles: 2-year follow-up Time 2 visit) and after controlling for total cortical surface area (black circles). Both sets of analyses included statistical controls for sex, age, parental education, research site, handedness, and Freesurfer topological defects. The horizontal line is the Cohen’s d score corresponding to a *p* < 0.05 effect after Bonferroni correction for 21 comparisons when considering the 7 toolbox measures and the 3 PTA measures. Executive function and working memory data were not available in the time 2 data set.

Figure 1 shows the distribution of Cohen’s *d* effect sizes for the receptive vocabulary (*x*-axis) and oral word reading (*y*-axis) associations across Destrieux region asymmetry measures after accounting for age, sex, parental education, handedness, research site, and the number of Freesurfer topological defects. These results show that surface area asymmetry effect sizes were similar across the receptive vocabulary and word reading measures. These results also show that medial PTA exhibited the largest association with receptive vocabulary and oral word reading compared to surface area asymmetries for the other Destrieux regions (see Supplementary Table S2). The receptive vocabulary and oral word reading effects were consistent across measurement time points and appeared to be due to the influence of total cerebral surface area on the medial PTA and cognitive measures (Figure 2; Supplementary Figure S2 shows the anterior and posterolateral PTA results).

### 3.2. Behavioral Specificity

Figure 2 shows that oral word reading, executive function, and episodic memory were significantly associated with medial PTA, in addition to receptive vocabulary. The specificity of these behavioral and medial PTA associations was examined with three different hierarchical regression models where receptive vocabulary predicted medial PTA in the first level of the models, and then oral word reading, executive function, or episodic memory were included in the second level of three separate regression models. Table 2 shows that children with better receptive vocabulary had significantly more leftward medial PTA after controlling for oral word reading. In contrast, oral word reading was no longer significantly associated with medial PTA when included in the regression model with receptive vocabulary (Supplementary Figure S3 shows the medial PTA associations with these measures). Receptive vocabulary also remained significantly associated with medial PTA when executive function or episodic memory measures were included in the regression model with medial PTA, as shown in Table 2. That is, receptive vocabulary exhibited a significant association with medial PTA when considering the potential influences of other NIH Toolbox Cognition measures.

### 3.3. Sex Effects

Girls and boys appeared to contribute to the medial PTA association with receptive vocabulary (girls: *t*_5504_ = 2.253, *p* = 0.024; boys: *t*_6024_ = 4.684, *p* = 2.88^−06^). While boys exhibited a larger t-score, examination of a sex-by-medial PTA interaction did not indicate a significantly stronger receptive vocabulary association for boys compared to girls (*t* = 1.303, *p* = 0.192). Girls did not exhibit a significant association between medial PTA and oral word reading (*t*_5498_ = 0.617, *p* = 0.537), in contrast to the boys (*t*_6016_ = 3.822, *p* = 0.0001). Here, the sex-by-medial PTA interaction was significant (*t*_11,547_ = 2.143, *p* = 0.032). This interaction effect was also observed in the 2-year follow-up data (*t*_7379_ = 2.358, *p* = 0.018). Thus, there were small effect size associations between medial PTA and receptive vocabulary across the sample, but only boys exhibited evidence of a small effect association between medial PTA and oral word reading.

## 4. Discussion

The results of this study demonstrate significant but small associations between PTA and language-related measures, with modestly larger effects for a receptive vocabulary measure compared to other cognitive constructs. More leftward PTA was also significantly and weakly related to better oral word reading, but only in boys. These results were replicable within the sample at different cross-sectional time points. Thus, a large sample size is necessary to reliably observe small effect PTA and language-related measure associations that are more consistent across language-related measures for boys than girls. If there is a direct contribution of PTA to language development, as described in the cerebral lateralization hypothesis, this effect appears to be modest based on the gross surface area asymmetries examined in the current study of children who were sampled with epidemiological practices [21] rather than with a targeted sampling of children who have oral and/or written language impairments.

The largest planum temporale effect size associations with the language-related measures were observed for a medial planum temporale region compared to anterior and posterolateral planum regions that are both highly variable in sulcal/gyral morphology [32], as well as in comparison to all other Destrieux cortical regions. In contrast to the anterior and posterolateral planum temporale regions, the medial planum region exhibited a rightward asymmetry across the sample. The medial PT typically exhibits a leftward voxel-based gray matter volume asymmetry across people [33]. The rightward asymmetry of this Destrieux region of interest may be explained by the inclusion of the medial parietal operculum within this medial planum temporale region of interest, thus highlighting one limitation of using the Destrieux atlas. However, more leftward medial PTA was related to: (1) better vocabulary performance; (2) increased total cortical surface area; and (3) more leftward posterolateral PTA, which also exhibited a modest association with receptive vocabulary (Supplementary Table S1).

The relatively stronger PTA association with receptive vocabulary compared to oral word reading indicates that variation in PT morphology relates more strongly to the acquisition of verbal knowledge than the ability to read real words. The smaller real word reading effect, due in part to a smaller association in girls, is difficult to interpret because the real word reading stimulus list includes words with regular and irregular orthography. Irregular or exception word reading ability appears to be better in children with deeper receptive vocabulary knowledge [34], perhaps due to top-down knowledge about how to pronounce and familiarity with irregular words [35]. Children with better receptive vocabulary may thus be less reliant on a phonological strategy for sounding out words that would negatively impact the ability to read exception words (e.g., words with identical spelling patterns but different pronunciations like few, sew) [36]. Children with shallow receptive vocabulary, including those with poor phonological decoding, may rely on a phonological strategy when trying to recognize irregular words and make more errors. This effect would be expected to be more pronounced in boys because girls appear to be less reliant on phonology for identifying words [36,37]. That is, the non-significant association between real word reading and PTA in girls compared to boys may reflect different influences on the ability to read irregular words between boys and girls, to the extent that irregular word reading contributed to the effect in boys. This may include expressive language and listening comprehension abilities that were not assessed in the current sample, and that can predict individual differences in oral word reading [38].

The association between PTA and receptive vocabulary, but not oral word reading, in girls also appears relevant for evaluating the cerebral lateralization hypothesis that testosterone underlies a PTA and language association. That is, the significant PTA association with receptive vocabulary in girls would not be expected if testosterone influences language development by impacting temporal lobe pruning early in development.

Advancing understanding about how cerebral lateralization influences language learning may depend on identifying subsets of participants in large epidemiological datasets with specific oral language learning profiles, given the otherwise modest effects across the sample. This subset of participants would be expected to have low total brain volume given that total cortical surface area appeared to impact the medial PTA results in this study (Figure 2) and because lower total brain volume is consistently related to language impairment and lower verbal ability [39,40,41]. These participants might also be expected to have evidence of ectopias, to the extent that ectopias can be observed with ultrahigh field MRI, given that brain weight appeared to decrease with increasing number of ectopias in those landmark post-mortem cases that provided an empirical foundation for the cerebral lateralization hypothesis [1].

There is another explanation for a PTA association with language-related abilities in other studies that is complementary to a brain size explanation. PTA and language-related associations may be due to a subset of people who experience disrupted neural development (e.g., cortical dysplasia) within brain regions that typically support language, thus producing a dependence on the right hemisphere to support language when they have rightward PTA. While it was not clear from the available data if there were ABCD participants with disrupted neural development, this hypothesis is supported by a study of children with left-hemisphere drug-resistant epilepsy who presumably had disrupted left-hemisphere language development and function. Children with more rightward PTA had atypical language organization and lower verbal ability [42]. That is, rather than non-leftward PTA reflecting aberrant development, this asymmetry may lead to inefficient language processing when other brain regions cannot effectively support language. Here, non-leftward PTA would occur with atypical reading and language abilities when there is atypical development of other language and reading network region(s), but non-leftward PTA alone would be insufficient to produce language impairments. For example, there may be an increased dependence on right hemisphere auditory cortex processing with atypical left superior temporal sulcus development, which typically supports auditory working memory [43] and has been linked to auditory working memory ability [44] and reading disability [41]. Thus, compensation and/or reorganization due to disrupted development within the reading [45] and/or language network [46], as well as a scaling of leftward PTA with brain size, could each theoretically contribute to PTA and language associations. Multiple mechanisms for PTA associations with reading and language performance could also explain why atypical PTA has been observed in people with phonological impairments when controlling for total brain volume [9], as well as the results of this study where PTA and language-related associations appeared to depend on total surface area.

## 5. Conclusions

Small effect size associations were observed between PTA and language-related measures that depended on the power of a large sample. These results also appeared to depend on a measure of verbal ability and were less consistent for a measure of oral word reading. The mechanism(s) underlying these associations are unclear from the current study. Advancing mechanistic understanding may require identifying subset(s) of children who most contribute to cerebral asymmetry and language-related associations.

## Figures and Tables

**Table 1:** Descriptive statistics for the NIH Toolbox Cognition and Freesurfer surface area asymmetries.

	Boys		Girls		Group Difference
	Mean	SD	Mean	SD	*p* Value
Oral Word Reading ^	90.879	7.101	90.837	6.701	
Receptive Vocabulary	84.705	8.063	84.191	8.170	***
Attention	94.165	9.348	93.817	0.0395	
Executive Function ^	92.002	9.957	93.094	8.965	***
Episodic Memory	102.088	12.032	103.610	12.070	***
Processing Speed ^	87.168	14.866	89.032	14.202	***
Working Memory	97.036	12.240	96.234	11.892	***
Medial PTA † ^	−0.174	0.124	−0.207	0.110	***
Anterior PTA †	0.291	0.263	0.299	0.253	
Posterolateral PTA ^	0.182	0.214	0.144	0.204	***
Total Cortical Surface Area ^	197,494	16,850	180,889	15,512	***

*Note*. There was no significant interaction between sex and PTA in predicting receptive vocabulary, and thus, results from the combined analysis of data from girls and boys are presented in the manuscript. Because of the modestly stronger effects in boys compared to girls, we present descriptive statistics for each group and group differences here and in the following table. † One-sample *t*-tests demonstrated significant rightward asymmetry (medial PTA) or significant leftward asymmetry (anterior and posterolateral PTA) across the sample. Note that while medial PTA exhibited rightward asymmetry across the boys and girls, children with more leftward asymmetry demonstrated better language-related performance. ^ Significantly increased variance in boys compared to girls based on the Levene test of variance after Bonferroni correction. *** Sex differences: *p* < 0.001.

**Table 2:** Three hierarchical multiple regressions show the relative strength of the receptive vocabulary association with medial PTA when oral word reading, executive function (card sorting), and episodic memory were included in the model, respectively.

Regression Model	NIH Toolbox Cognition Variable	Estimate	Standard Error	*t*-Score	
Level 1	Receptive Vocabulary	7.80^−4^	1.55^−4^	5.041	***
Model 1:	Receptive Vocabulary	6.69^−4^	1.71^−4^	3.905	***
Level 2	Oral Word Reading	2.78^−4^	1.92^−4^	1.446	
Model 2:	Receptive Vocabulary	6.99^−4^	1.58^−4^	4.429	***
Level 2	Executive Function	3.15^−4^	1.24^−4^	2.541	*
Model 3:	Receptive Vocabulary	7.21^−4^	1.57^−4^	4.599	***
Level 2	Episodic Memory	2.41^−4^	9.48^−5^	2.546	*

*Note*. The regression models for the results shown here included sex, age, parental education, handedness, research site, and Freesurfer topological defects as control variables. Behavioral control variables effects when included in the first level of the regression instead of receptive vocabulary are as follows: oral word reading *t* = 3.466, *p* = 0.0005; executive function: *t* = 3.509, *p* = 0.0005; episodic memory: *t* = 3.406, *p* = 0.0007. *** *p* < 0.001; * *p* < 0.05.

## Data Availability

The specific ABCD data used in this study is available at doi: 10.15154/1528299.

## Supplementary Materials

The following supporting information can be downloaded at https://www.mdpi.com/article/10.3390/brainsci14010014/s1. Figure S1: Planum temporale regions of interest, their surface area asymmetry distributions, and inter-relationships; Figure S2: Effect sizes for the relationship between baseline PTA and NIH Toolbox Cognition measures is shown before and after controlling for total cortical surface area; Figure S3: More leftward medial PTA occurred with (A) better receptive vocabulary and (B) better oral word reading. Positive values indicate more leftward asymmetry. SSU: standardized scores uncorrected for age. Bivariate correlations coefficients and regression results are presented in Section 3.1; Table S1: Pearson correlations between demographic, receptive vocabulary and oral word reading, and brain structure measures; Table S2: Effect sizes for the association between each Destrieux region surface area asymmetry and the NIH Toolbox language-related measures.
