# Supplementary material for: Auditory Cortex Asymmetry Associations with Individual Differences in Language and Cognition
Source: Brain Sci. 2023 Dec 23;14(1):14. doi: 10.3390/brainsci14010014 (PMC10813516; doi:10.3390/brainsci14010014)
Supplement: Supplementary file 1 [file brainsci-14-00014-s001.zip › brainsci-2763387-supplementary.pdf]

brain sciences

MDPI

Auditory Cortex Asymmetry Associations with Individual Differences in Language and Cognition

Mark A. Eckert [1,*], Kenneth I. Vaden, Jr. [1] and Silvia Paracchini [2]

[1] Department of Otolaryngology—Head and Neck Surgery, Medical University of South Carolina, Charleston, SC 29425, USA; vaden@musc.edu
[2] School of Medicine, University of St. Andrews, North Haugh, St. Andrews KY16 9TF, UK; sp58@st-andrews.ac.uk
* Correspondence: eckert@musc.edu

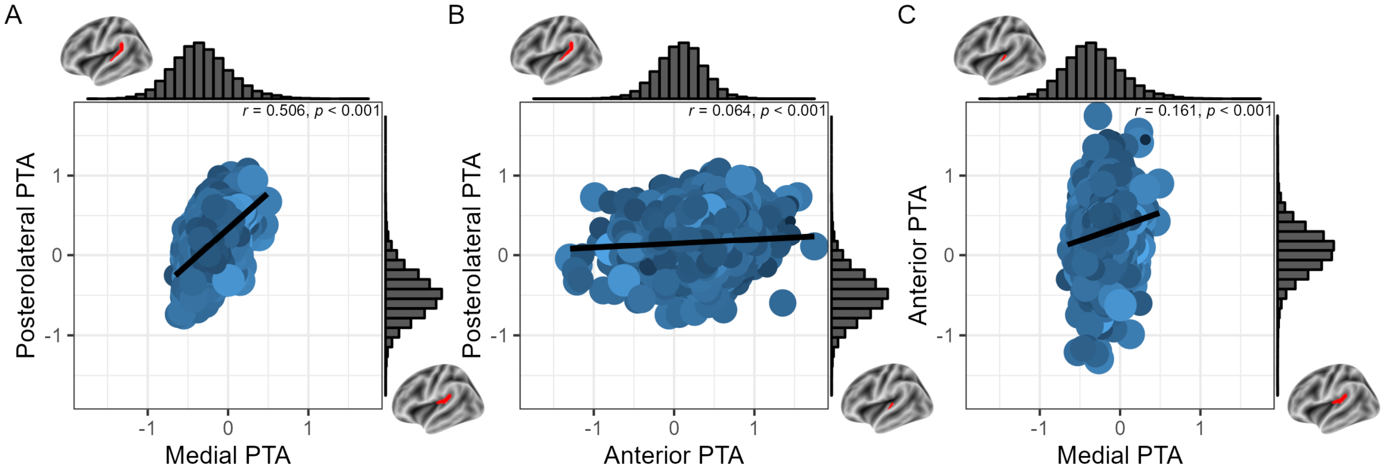

Figure S1. Planum temporale regions of interest, their surface area asymmetry distributions, and inter-relationships. A) The medial PTA and posterolateral PTA variables were more strongly associated than B,C) the anterior (Heschl's sulcus) PTA with the medial and posterolateral PTA. The scale of each axis denotes asymmetries that are defined as (left – right)/ ((left + right)/2), where positive values indicate leftward asymmetries. Lighter color shading and larger symbol size reflects a larger total cortical surface area.

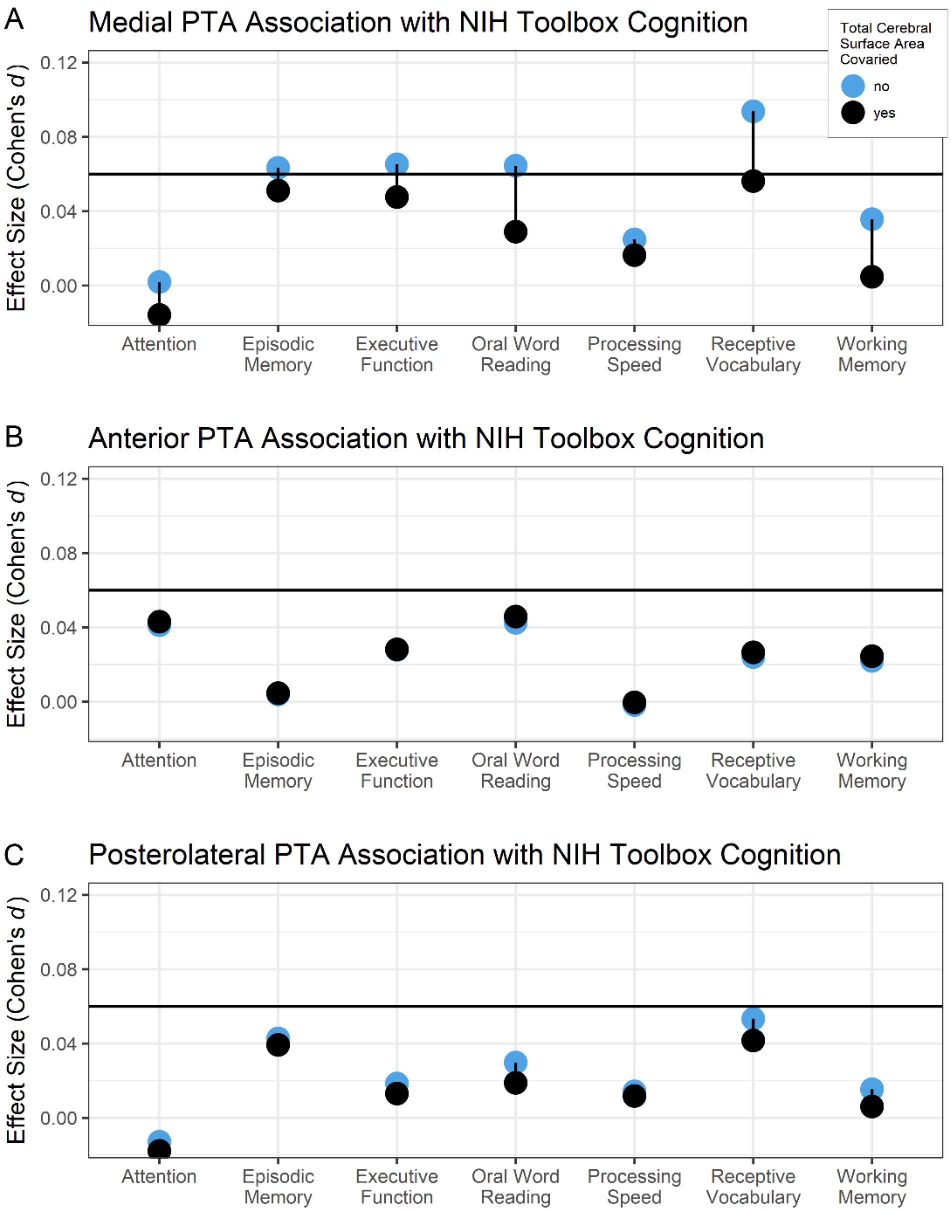

Figure S2. Effect sizes for the relationship between baseline PTA and NIH Toolbox Cognition measures is shown before (blue) and after (black) controlling for total cortical surface area for the medial PTA (**A**), anterior PTA (**B**), and posterolateral PTA (**C**). Both sets of analyses included statistical controls for sex, age, parental education, research site, and Freesurfer topological defects. The horizontal line is the small effect Cohen's *d* score corresponding to a $p < 0.05$ effect after Bonferroni correction for 21 comparisons given the large ABCD sample size.

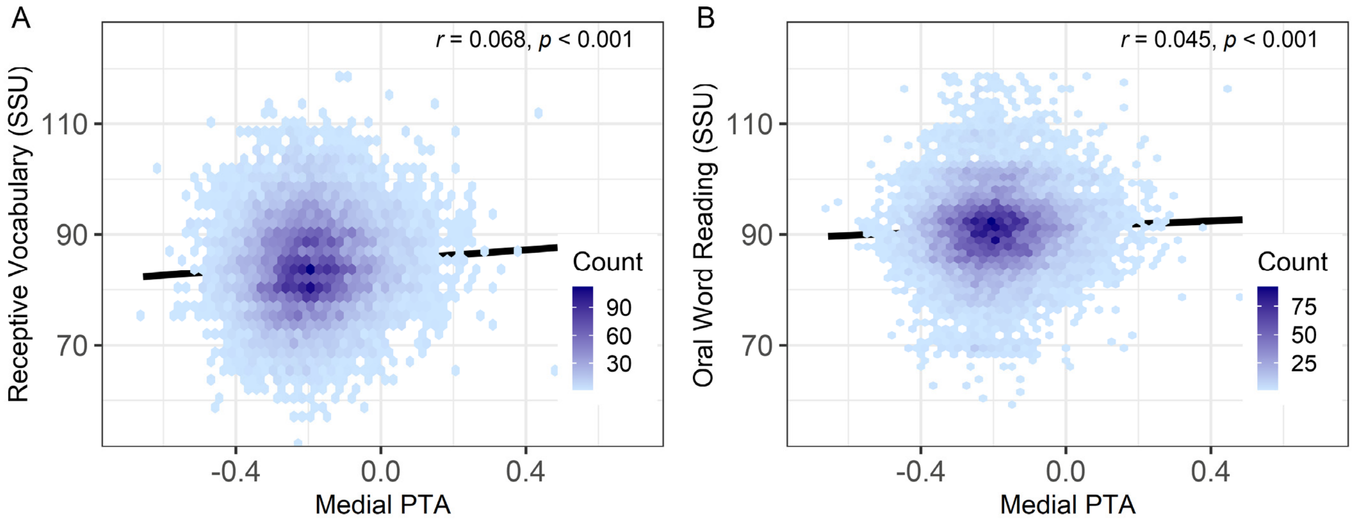

Figure S3. −More leftward medial PTA occurred with (**A**) better receptive vocabulary and (**B**) better oral word reading. Positive values indicate more leftward asymmetry. SSU: standardized scores uncorrected for age. Bivariate correlations coefficients and regression results are presented in Section 3.1.

Table S1. Pearson correlations between demographic, receptive vocabulary and oral word reading, and brain structure measures.

Variable	1	2	3	4	5	6	7	8	9
1. Sex (Boys:1; Girls:2)									
2. Age	−0.02* [−0.04, −0.00]								
3. Parental Education	−0.01 [−0.02, 0.01]	0.02* [0.00, 0.04]							
4. Handedness (Non−Right:0; Right:1)	−.02* [−0.04, −0.00]	−.01 [−0.03, 0.01]	.01 [−0.01, 0.03]						
5. Receptive Vocabulary	−0.03** [−0.05, −0.01]	0.23** [0.22, 0.25]	0.41** [0.39, 0.42]	0.00 [−0.02, 0.02]					
6. Oral Word Reading	−0.00 [−0.02, 0.02]	0.22** [0.20, 0.23]	0.33** [0.32, 0.35]	−0.02 [−0.04, 0.00]	0.53** [0.52, 0.55]				
7. Medial PTA	−0.14** [−0.16, −0.12]	−0.00 [−0.02, 0.02]	0.06** [0.04, 0.07]	−0.01 [−0.02, 0.01]	0.07** [0.05, 0.09]	0.04** [0.03, 0.06]			
8. Anterior PTA	0.02 [−0.00, 0.03]	0.01 [−0.01, 0.03]	0.00 [−0.02, 0.02]	−0.01 [−0.03, 0.01]	0.02* [0.00, 0.04]	0.02** [0.01, 0.04]	0.16** [0.14, 0.18]		
9. Posterolateral PTA	−0.09** [−0.11, −0.07]	0.01 [−0.01, 0.03]	0.03** [0.01, 0.05]	−0.01 [−0.03, 0.01]	0.04** [0.02, 0.06]	0.02* [0.00, 0.04]	0.51** [0.49, 0.52]	0.06** [0.05, 0.08]	
10. Total Surface Area	−0.46** [−0.47, −0.44]	−0.01 [−0.03, 0.01]	0.19** [0.17, 0.20]	0.00 [−0.01, 0.02]	0.23** [0.21, 0.24]	0.19** [0.17, 0.21]	0.16** [0.14, 0.18]	−0.01 [−0.03, 0.01]	0.07** [0.05, 0.09]

Posterolateral PTA did not exhibit a significant association with receptive vocabulary after controlling for medial PTA (t = 0.404, p = 0.686).

Table S2. Effect sizes for the association between each Destrieux region surface area asymmetry and the NIH Toolbox language-related measures.

Destrieux (label) Asymmetry Region of Interest (ordered by descending Cohen's *d* for Receptive Vocabulary Knowledge)	Cohen's *d*	
	Oral Word Reading	Receptive Vocabulary Knowledge
posterior ramus of the lateral sulcus (medial PTA)	0.089	0.118
anterior segment of the circular sulcus of the insula	0.023	0.081
subcallosal gyrus	0.069	0.080
pericallosal sulcus	0.057	0.072
planum temporale (posterolateral PTA)	0.046	0.068
opercular part of the inferior frontal gyrus	0.046	0.061
planum polare of the superior temporal gyrus	0.031	0.060
paracentral lobule and sulcus	0.030	0.056
long insular gyrus and central sulcus of the insula	0.032	0.051
anterior transverse collateral sulcus	0.047	0.049
lateral orbital sulcus	0.060	0.047
supramarginal gyrus	0.035	0.045
superior segment of the circular sulcus of the insula	0.006	0.039
triangular part of the inferior frontal gyrus	0.018	0.039
posterior-dorsal part of the cingulate gyrus	0.004	0.037
lateral aspect of the superior temporal gyrus	0.042	0.037
middle-anterior part of the cingulate gyrus and sulcus	−0.004	0.035
inferior frontal sulcus	0.019	0.035
anterior part of the cingulate gyrus and sulcus	0.037	0.032
inferior part of the precentral sulcus	0.055	0.029
lateral occipito-temporal gyrus	−0.011	0.028
transverse temporal sulcus (anterior PTA)	0.045	0.027
subcentral gyrus and sulci	−0.001	0.026
orbital gyri	0.011	0.024
temporal pole	0.020	0.022
central sulcus	0.028	0.018
middle frontal gyrus	0.050	0.018
superior occipital sulcus and transverse occipital sulcus	0.060	0.018
transverse frontopolar gyri and sulci	−0.001	0.012
superior frontal sulcus	0.006	0.012
superior occipital gyrus	−0.008	0.009
postcentral gyrus	0.027	0.008
lateral occipito-temporal sulcus	0.002	0.008
cuneus	−0.006	0.008
anterior transverse temporal gyrus	0.003	0.006
gyrus rectus	0.034	0.004
horizontal ramus of the anterior segment of the lateral sulcus	−0.001	0.003
middle-posterior part of the cingulate gyrus and sulcus	−0.049	0.002
orbital part of the inferior frontal gyrus	−0.003	0.002
precentral gyrus	0.004	−0.001
marginal branch of the cingulate sulcus	0.017	−0.001
parahippocampal gyrus	0.032	−0.001
inferior temporal gyrus	0.019	−0.003
fronto−marginal gyrus and sulcus	−0.007	−0.003
subparietal sulcus	−0.016	−0.004
vertical ramus of the anterior segment of the lateral sulcus	0.019	−0.004
medial occipito−temporal sulcus and lingual sulcus	−0.011	−0.006
posterior transverse collateral sulcus	−0.007	−0.007
middle frontal sulcus	−0.027	−0.008
superior part of the precentral sulcus	−0.042	−0.009
anterior occipital sulcus and preoccipital notch	−0.030	−0.013
calcarine sulcus	−0.015	−0.015
inferior segment of the circular sulcus of the insula	0.015	−0.016
posterior−ventral part of the cingulate gyrus	−0.014	−0.017

lingual gyrus	−0.003	−0.018
intraparietal sulcus and transverse parietal sulci	−0.025	−0.018
postcentral sulcus	−0.009	−0.020
sulcus intermedius primus	−0.048	−0.021
middle temporal gyrus	−0.024	−0.026
superior parietal lobule	−0.012	−0.028
occipital pole	−0.019	−0.030
inferior occipital gyrus and sulcus	−0.065	−0.031
short insular gyri	−0.031	−0.031
middle occipital gyrus	−0.008	−0.032
suborbital sulcus	−0.050	−0.033
superior temporal sulcus	−0.033	−0.043
medial orbital sulcus	−0.039	−0.044
angular gyrus	−0.018	−0.045
middle occipital sulcus and lunatus sulcus	−0.019	−0.047
inferior temporal sulcus	−0.008	−0.047
superior frontal gyrus	−0.037	−0.048
orbital sulci	−0.042	−0.063
parieto−occipital sulcus	−0.061	−0.070
precuneus	−0.024	−0.075

Negative effect sizes indicated a negative association between the asymmetry and behavioral measure. Blue font is used to highlight the PTA regions of interest.
